# Unraveling chromosomal and genotoxic damage in individuals occupationally exposed to coal from underground mining

**DOI:** 10.3389/fgene.2024.1422938

**Published:** 2024-07-04

**Authors:** María Yolanda Buitrago-Rodríguez, Nelson Rangel, Juan D. Vega-Valderrama, Martín Pulido-Medellín, Milena Rondón-Lagos

**Affiliations:** ^1^ School of Biological Sciences, Universidad Pedagógica y Tecnológica de Colombia, Tunja, Colombia; ^2^ Departamento de Nutrición y Bioquímica, Facultad de Ciencias, Pontificia Universidad Javeriana, Bogotá, Colombia; ^3^ Grupo de Investigación en Medicina Veterinaria y Zootecnia, Facultad de Ciencias Agropecuarias, Universidad Pedagógica y Tecnológica de Colombia, Tunja, Colombia

**Keywords:** underground mining, chromosomal alterations, micronuclei, chromosome instability, cytogenetic studies

## Abstract

**Purpose:**

Coal mining is a vital sector in Colombia, contributing significantly to the nation’s economy and the development of its regions. However, despite its importance, it has led to a gradual decline in the health of mine workers and nearby residents. While the adverse health effects of open-pit coal mining on exposed individuals have been well-documented in Colombia and globally, studies investigating genetic damage in underground coal miners are lacking.

**Methods:**

The aim of our study was to evaluate chromosomal and genotoxic damage, in peripheral blood samples from a group of underground coal miners and residents of areas exposed to coal, in the town of Samacá, Boyacá-Colombia, and in a group of unexposed individuals by using banding and molecular cytogenetic techniques, as well as cytokinesis block micronucleus assays.

**Results:**

Our results suggest that occupational exposure to coal induces chromosomal and genotoxic damage in somatic cells of underground coal miners. Chromosomal and genotoxic damage is an important step in carcinogenesis and the development of many other diseases. Our findings provide valuable insights into the effects of coal dust exposure on chromosomal integrity and genetic stability.

**Conclusion:**

Our pilot study suggests that occupational exposure to coal induces chromosomal damage in underground coal miners, highlighting the importance of validating these findings with a larger sample size. Our results highlight the need to implement prevention and protection measures, as well as educational programs for underground coal miners. Characterizing and estimating exposure risks are extremely important for the safety of people exposed occupationally and environmentally to coal and its derivatives.

## 1 Introduction

Coal mining plays a pivotal role in Colombia’s economy and the development of its producing regions, contributing significantly to the national energy matrix (Bustamante Ortega et al., 2021). In our country, coal extraction occurs through both, open-pit and underground methods. However, despite its significance, this industry has led to a gradual decline in the health of mining workers and residents of surrounding areas (Álvarez Sánchez et al., 2016; Ayala Mosquera et al., 2019). This is attributed to exposure to coal dust, which contains mineral particles, inorganic compounds, polycyclic aromatic hydrocarbons, and ionizing radiation generated throughout the extraction, transportation, utilization, and combustion processes of the mineral (Álvarez Sánchez et al., 2016; Acosta Bueno and Novoa Patiño, 2017). These factors can induce oxidative stress and promote inflammation that leads to DNA damage (Sinitsky et al., 2016).

While the detrimental effects on the health of individuals exposed to open-pit coal mining have been well-documented in Colombia and globally (Rudas and Hawkins, 2014; Espitia-Pérez et al., 2018; Ayala Mosquera et al., 2019), research examining chromosomal and genotoxic damage in underground coal miners is notably lacking or scarce. This is particularly evident in regions like Samacá, Boyacá-Colombia, where underground mining operations are prevalent, leading to heightened occupational risks, accidents, and diseases compared to areas with open-pit mining activities (Rudas and Hawkins, 2014). Indeed, in 2016, reports surfaced regarding the prevalence of cardiovascular ailments such as cardiac arrhythmias, acute myocardial infarction, and heart failure, alongside respiratory conditions like asthma, infant mortality, and lung cancer among residents in the mining enclaves of Samacá (Acosta Bueno and Novoa Patiño, 2017). The above underscores the need to deepen and expand our understanding of chromosomal and genotoxic damage resulting from coal exposure in underground mines. These efforts are crucial to establish future applications of early diagnostic testing and the establishment of follow-up programs aimed at mitigating coal mining-induced adverse impacts on the exposed population. Considering the above, the aim of our study was to evaluate the chromosomal and genotoxic damage, in peripheral blood samples from a group of underground coal miners and residents of areas exposed to coal, in the town of Samacá, Boyacá-Colombia, and in a group of unexposed individuals (control group), by using banding and molecular cytogenetic techniques, as well as cytokinesis block micronucleus assays. Characterizing and estimating exposure risks are extremely important for the safety of people exposed occupationally and environmentally to coal and its derivatives.

## 2 Materials and methods

### 2.1 Study population

A total of 20 individuals were part of this study, which were divided into two study groups: the first group included five (5) underground coal miners (M), occupationally exposed to coal, who develop their activity in a mine in the town of Samacá, Boyacá, and five (5) unexposed control individuals (MC), from the same geographic region, age, and sex, with no history of occupational exposure to genotoxic agents such as coal, chemicals, radiation, or cigarettes (Sinitsky et al., 2016). The group of underground coal miners (M), consisted of men between 40 and 52 years old, involved in coal mining for at least 120 months. The unexposed control group (MC) consisted of healthy men, without indication of previous occupational exposure to coal, and with an age range (between 40 and 53 years), distribution by sex and life habits similar to the exposed group (Table 1A; Supplementary Table S1A). The group of underground coal miners analyzed in this study were all male, due to the fact that this work activity is mainly carried out by men (González et al., 2017), who perform the function of pikemen, which consists of exploiting the coal seam, adopting forced postures with reduced space in their workplace, being considered the occupation more frequent in mining (Jiménez-Forero et al., 2015).

**TABLE 1:** General characteristics of the groups studied.

	M	MC	R	RC
Number	5	5	5	5
Age (mean ± SD)	45.4+/−5.3	45+/−5.7	47.6+/−13.6	46.8+/−14.1
Sex (n)				
Male	5	5	0	0
Female	0	0	5	5
Exposure months (mean ± SD)	204+/−77.3	0	288+/−181.6	0
Smoke (n)				
Smokers	0	0	0	0
Non-smokers	5	5	5	5
Alcohol (n)				
Drinkers	3	5	2	2
Non-drinkers	2	0	3	3

**Abbreviations:** M, exposed miner; MC, unexposed control group; R, exposed resident; RC, unexposed control group; SD, standard deviation.

The second group included, five (5) residents (R), environmentally exposed (indirectly) to coal, who do not work in the mines, but who live in areas close to it, and five (5) control individuals (RC), from the same geographic region, age, and sex, with no history of occupational exposure to genotoxic agents such as coal, chemicals, radiation, or cigarettes (Sinitsky et al., 2016). The group of exposed residents (R), consisted of women between 25 and 61 years old, environmentally exposed to coal for at least 120 months. The unexposed control group (RC) consisted of healthy women, without indication of previous environmentally exposure to coal, and with an age range (between 23 and 60 years old), distribution by sex and life habits similar to the exposed group (Table 1; Supplementary Table S1B).

The study was conducted in accordance with the Declaration of Helsinki and approved by the Ethics Committee of Universidad Pedagógica y Tecnológica de Colombia, Tunja (date of approval June 13–2022). Written informed consent was obtained from each study participant.

Each participant was also required to complete a standardized questionnaire aimed at documenting potential confounding variables, including medical history, age, gender, smoking and drinking habits, duration and frequency of coal exposure, recent illnesses, and medical treatments (Table 1; Supplementary Table S1). Regarding employment history, participants were asked about the year of initial coal exposure and the duration of exposure. Exposure duration was defined as the period (measured in months) from the first exposure to coal in an occupational (miners) or environmental (residents) context until a change in workplace or cessation of work. Individuals with a history of cancer or undergoing prolonged medical treatment such as radiotherapy or chemotherapy were excluded from the study. Data from exposed individuals (M and R) were compared with those from unexposed individuals (MC and RC).

### 2.2 Samples collection

Five millilitres of peripheral blood, from exposed and unexposed individuals, were collected into heparinized tubes by venous puncture. The written informed consent of each subject participating in the study was obtained before the blood samples were taken.

### 2.3 GTG-banding cytogenetics

The metaphases and interphase nuclei of the cultured peripheral blood lymphocytes were obtained using standard protocols. Briefly, lymphocyte cultures were performed by adding 1 mL of whole blood, in 5 mL of RPMI−1640 medium (Sigma, St. Louis, MO, USA), supplemented with 10% fetal bovine serum (FBS) (Sigma) and 100 μL of phytohemagglutinin-M (Gibco, Life Technologies, Nebraska, USA). The cultures were incubated at 37°C for 72 h in a 5% CO_2_ atmosphere. After 72 h, a solution of N-deacetyl-N-methyl colchicine (0.0001 g/mL final concentration) (Sigma) was added to the cultures for 25 min. After this time, the cells were treated with hypotonic solution (KCl, 0.075 M), fixed with carnoy fixative (3:1 methanol: acetic acid), and spread on glass.

A portion of the metaphase and nuclear spreads obtained previously, was utilized to assess chromosomal alterations through GTG banding, while the remainder was allocated for Fluorescence in situ hybridization (FISH) assays. For GTG banding, chromosome spreads underwent incubation with trypsin (0.25%) (Gibco) and were subsequently stained with Giemsa (Sigma). All cultures of each individual, exposed and unexposed, were performed in duplicate.

Metaphase spreads were analyzed using an Olympus microscope and processed using the cytogenetic software Cytovision System 7.4 (Leica Biosystems Richmond, VA, USA). Chromosomal variants (CVs) [variation in length of heterochromatic segments on the long arms of chromosomes 1 (1qh+) and 9 (9qh+)], fragilities (fra), chromosomal breaks (chrb) and chromatid breaks (chrb), and chromosomal aberrations (CAs) including structural (SCAs) and numerical chromosomal aberrations (NCAs) were evaluated. All CVs and CAs were described according to the International System for Human Cytogenomic Nomenclature (ISCN) 2020 (Nomenclature and On, 2020).

### 2.4 Cytokinesis block micronucleus (CBMN) assay

The CBMN assay was performed using the protocol described by Fenech (2007). Briefly, lymphocyte cultures were performed by adding 1 mL of whole blood, in 5 mL of RPMI−1640 medium (Sigma), supplemented with 10% fetal bovine serum (FBS) (Sigma) and 100 μL of phytohemagglutinin-M (Gibco). The cultures were incubated at 37°C for 44 h in a 5% CO_2_ atmosphere. After 44 h, a solution of 5 μg/mL of cytochalasin B (Sigma) was added to the cultures for a total time of 72 h, as previously described (Fenech, 1993). After this time, the cells were treated with hypotonic solution (KCl, 0.075 M) for 8 min, and fixed with carnoy fixative (3:1 methanol: acetic acid). Thus obtained, the cell pellets were spread on glass slides and subsequently stained with 5% (v/v) Giemsa for 12 min. All cultures of each individual, exposed and unexposed, were performed in duplicate.

The presence of micronuclei (MN), nucleoplasmic bridges (NPB), and nuclear buds (NBUD), for each blood sample, was determined in total of 1,000 binucleated cells per individual (miners, residents, and their respective controls), photographed, and analyzed, using an Olympus brand microscope and the Cytovision System software 7.4 (40x magnification) (Leica Biosystems Richmond, Inc). The evaluation criteria described by Fenech, et al. (Fenech et al., 2003) for the identification of MN, NPB and NBUD was applied.

### 2.5 Evaluation of chromosomal instability and clonal heterogeneity

Chromosomal instability (CIN) was assessed on the nuclei spreads previously obtained, from exposed and unexposed individuals, by using FISH. Five centromere enumeration probes (CEP; Cytocell, Cambridge, UK) for chromosomes 2, 3, 11, 15, and 17 were employed. Dual-color FISH was performed for chromosomes 2 (CEP2; *Spectrum Orange*) and 11 (CEP11; *Spectrum Green*), as well as, for chromosomes 3 (CEP3; *Spectrum Orange*) and 15 (CEP15; *Spectrum Green*). For chromosome 17 (CEP17; *Spectrum Green*), single FISH was performed. FISH was performed following standard procedures. Ten random areas of nuclei spreads were acquired using an Olympus microscope with the cytogenetic software Cytovision System 7.4 (Leica Biosystems Richmond, Inc.)

CIN was evaluated in a minimum of 100 intact and non-overlapping nuclei per individual (miners, residents, and their respective controls). The CIN rate for each exposed and unexposed individual, was determined by first calculating, for each individual chromosome, the percentage of nuclei with a centromeric probe (CEP) signal number differing from the most common chromosome number (modal number). Subsequently, the mean CIN percentage of all analyzed chromosomes was computed (Lengauer et al., 1997; Munro et al., 2012). According to the level of CIN, each exposed and unexposed individual was categorized as having low CIN (CIN<25%) or high CIN (CIN≥25%) (Kawauchi et al., 2010; Talamo et al., 2010). The levels of CIN observed in each exposed individual were compared to those of the unexposed group.

To assess clonal heterogeneity (CH) and identify cell populations with varying levels of aneuploidy within each exposed and unexposed individual, we determined the true diversity index (TD) for all analyzed chromosomes (2, 3, 11, 15, and 17). TD integrates both, the quantity and distribution of cell populations within each individual, following established methodologies (Jost, 2006; Maley et al., 2006; Roylance et al., 2011). Based on the level of chromosomal heterogeneity (CH), each exposed and unexposed individual was categorized as having low CH (<1.5), intermediate CH (1.5≤CH < 2), or high CH (CH ≥ 2).

### 2.6 Statistical analysis

Fisher’s exact tests, Student’s t-tests, and unpaired Mann-Whitney Wilcoxon tests were conducted to compare the data obtained from GTG-banding cytogenetics and FISH between the exposed and unexposed groups, considering both parametric and non-parametric distributions. The normality and homoscedasticity of the data were evaluated using the Shapiro-Wilk test and Bartlett’s test, respectively. To investigate the potential associations between the frequency of CAs, CVs, CIN, MN, NPB, and NBUD, with variables such as age and exposure time to coal (only in exposed individuals), a multivariate analysis was conducted using the Pearson correlation coefficient. In addition, to confirm the effects of occupational exposure, a multiple regression analysis (multiple linear regression model) was also conducted.

The data from exposed individuals were compared with those from unexposed individuals. All statistical analyses were carried out using R Studio version 4.0.2, and *p* values < 0.05 were considered statistically significant (*p ≤ 0.05, **p ≤ 0.01, and ***p ≤ 0.001).

## 3 Results

### 3.1 Characteristics of study groups

The average duration of coal exposure among the exposed miners (M) group was 204 months, with an average age of 45.4 years (Table 1). It is worth highlighting that all individuals included in the study from the underground coal miners’ group were male. This reflects the predominant male participation in this type of work activity (González et al., 2017), particularly in roles like pikemen, which involve working in confined spaces with physically demanding postures, and is considered one of the most common occupations in mining (Jiménez-Forero et al., 2015). Regarding the group of exposed residents (R), the mean time of exposure to coal was 288 months and the mean age was 47.5 years (Table 1).

A low prevalence of alcohol consumption and absence of cigarette consumption was reported in all groups, exposed (M and R) and unexposed (MC and RC). The results are expressed as the mean ± standard deviation (SD) (Table 1; Supplementary Table S1).

### 3.2 Exposed miners and residents exhibit high chromosomal damage

GTG banding cytogenetic analysis was conducted on both, the exposed (M and R) and unexposed (MC and RC) groups, revealing a modal diploid number (2n). A total of 581 metaphases were analyzed, with each group ranging from 11 to 45 metaphases exhibiting good chromosome morphology and dispersion. Following the International recommendations for constitutional study analysis (CCMG-CCGM National Office, 2023; Ozkan and Marcelo Lacerda, 2023), we examined a minimum of 11 metaphases in cases where no NCAs or SCAs were detected, across all groups (exposed and unexposed). For cases where NCAs or SCAs were identified, we expanded the cytogenetic analysis to a maximum of 45 metaphases. The variation in the number of metaphases analyzed also reflects differences in the mitotic index among individuals included in the study.

**FIGURE 1:**
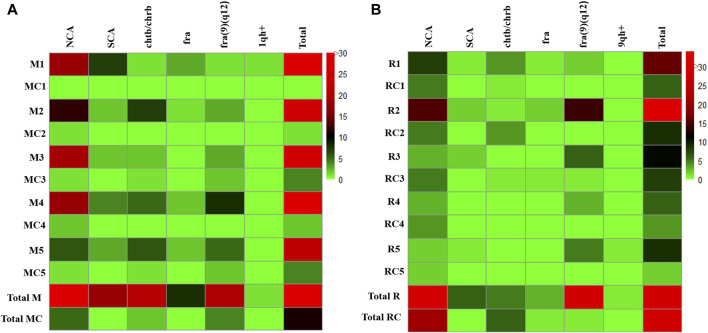
Total chromosomal variants and chromosomal aberrations observed in **(A)** Exposed Miners and Miner control group (unexposed control group), and in **(B)** Exposed Residents and Resident Control group (unexposed control group). Each row in the figure, represents a participant in the study. The frequency of each chromosomal variant and chromosomal aberration is indicated for each individual using a color code on the right. Abbreviations: M, Exposed Miner; MC, Miner control (unexposed control group); R, Exposed Resident; RC, Resident control (unexposed control group); NCAs, numerical chromosomal alterations; SCAs, structural chromosomal alterations; chtb, chromatidic break; chrb, chromosomic break; fra, fragilities; fra (9) (q12), fragility in the long arm of chromosome 9, region 1 and band 2; 1qh+, heterochromatin increased on long arm of chromosome 1; 9qh+, heterochromatin increased on long arm of chromosome 9.

Significantly high frequencies of CVs and CAs (NCAs, SCAs, chrb, chrb and fra), were found in the group of exposed miners (M) compared with those observed in the unexposed control group (MC) (141 and 11, respectively) (*p* ≤ 0.0093**; unpaired Mann Whitney Wilcoxon test) (Table 2; Figure 1).

**TABLE 2:** Chromosomal variants and chromosomal aberrations identified in Exposed and unexposed control groups.

CVs and CAs	Number of alterations			Number of individuals			Number of alterations			Number of individuals		
	M n (%)	MC n (%)	p+	M n (%)	MC n (%)	p+	R n (%)	RC n (%)	p+	R n (%)	RC n (%)	p+
Monosomies	39 (20.1)	3 (3.7)	0.0008**	5 (100)	3 (60)	0.444	24 (13.1)	7 (5.6)	0.1464	4 (80)	4 (80)	1
Trisomies	34 (17.5)	2 (2.5)	0.0008**	5 (100)	2 (40)	0.166	7 (3.8)	14 (11.2)	0.1046	3 (60)	5 (100)	0.4444
SCAs	18 (9.2)	0 (0)	0.0032**	5 (100)	0 (0)	0.007**	6 (3.3)	0 (0)	0.2462	4 (80)	0 (0)	0.0476*
chtb/chrb	21 (10.8)	2 (2.5)	0.0489*	5 (100)	2 (40)	0.166	5 (2.7)	6 (4.8)	0.7209	2 (40)	3 (60)	1
fra	8 (4.1)	0 (0)	0.1212	4 (80)	0 (0)	0.047*	3 (1.6)	1 (0.8)	1	2 (40)	1 (20)	1
fra (9)(q12)	20 (10.3)	4 (5.0)	0.2828	5 (100)	2 (40)	0.166	30 (16.5)	1 (0.8)	0.0001**	5 (100)	1 (20)	0.0476*
1qh+	1 (0.5)	0 (0)	1	5 (100)	2 (40)	1	0 (0)	0 (0)	1	0 (0)	0 (0)	1
9qh+	0 (0)	0 (0)	1	0 (0)	0 (0)	1	1 (0.6)	0 (0)	1	1 (20)	0 (0)	1
Total	141	11					76	29				
Mean	20	1.57					15.2	5.8				
SD	13.33	1.61					11.33	5.2				
p++	0.0093**						0.3122					

**Notes:** *Statistically significant difference relative to unexposed control group at *p* ≤ 0.05. **Statistically significant difference relative to unexposed control group at *p* ≤ 0.01 (p^+^: Fisher’s exact test; p^++^: unpaired Mann Whitney Wilcoxon test). Abbreviations: CVs, Chromosomal variants; CAs, chromosomal aberrations; M, exposed miner; MC, Miner control (unexposed control group); R, exposed resident; RC, Resident control (unexposed control group); SCAs, structural chromosomal alterations; chtb, chromatidic break; chrb, chromosomic break; fra, fragilities; fra(9)(q12), fragility in the long arm of chromosome 9, region 1 and band 2; 1qh+, heterochromatin increased on long arm of chromosome 1; 9qh+, heterochromatin increased on long arm of chromosome 9.

Specifically, in the group of exposed miners (M), a total of 194 metaphases were analyzed. In the analyzed metaphases, were observed: 73 NCAs, 18 SCAs, 21 chtb/chrb, 28 fragilities and 1 chromosomal heteromorphisms (1qh+). It is noteworthy that the presence of 1qh+ was detected in only 1 (20%) individual, whereas NCAs, SCAs, chtb/chrb, and fragilities were observed in all individuals (100%) (Table 2). Among the NCAs, the monosomies (53.42%) were more frequent than trisomies, polyploidies and endoreduplications (46.57%). The chromosomes with the highest frequency of monosomies were, the chromosome Y (12.82%), observed in 2 exposed miners (40%) and chromosome 22 (10.25%), observed in 3 (60%) exposed miners (Table 2). Within the trisomies, marker chromosomes were observed in greater frequency (41.17%) in all exposed miners (100%), followed by polyploidies (11.76%), observed in 3 (60%) exposed miners, and trisomy of chromosome 13 (8.8%) observed in 2 exposed miners (40%) (Table 2).

Regarding SCAs, a total of 18 SCAs were observed in the 100% of the exposed miners (Figure 1A; Table 2), being the most frequent the deletions (del) (50%), followed by derivatives chromosomes (der) (16.6%), additional material of unknown origin (add) (11.1%) and inversions (inv) (11.1%). Other structural alterations observed less frequently included, translocations (t) (5.5%) and telomeric associations (tas) (5.5%). The chromosomes most frequently involved in SCAs were chromosomes X, 1, 3, 7, 9, 12 and 18. Among SCAs, del (18)(p11), was observed commonly in two exposed miners (40%). Regarding chtb/chrb, these were observed in high frequency (21 ruptures) in all exposed miners (100%). Among chromatidic breaks, chtb (9)(q12) was the most frequent (14.28%), followed by chtb (1)(p32) and chtb (1)(q12), which were observed in more than one (1) exposed. Fragilities (fra) were also observed in the group of exposed miners (28 fragilities) (Figure 1A), being the most frequent the fra (9)(q12) (71.4%) observed in all exposed miners (100%).

In the unexposed control group (MC), a total of 80 metaphases were analyzed, being identified: 5 NCAs in 4 (60%) unexposed individuals; 2 chtb/chrb in 2 (40%) unexposed individuals and 4 fragilities in 2 (40%) unexposed individuals. No SCAs or chromosomal heteromorphisms were observed in this group (MC). Among the NCAs, the monosomies (60%) were more frequent than trisomies (40%). The chromosomes that presented monosomies were the chromosome 15 (66.6%) observed in 2 unexposed (40%) and chromosome 18 (33.3%) observed in 1 (20%) unexposed individual. Within the trisomies, only marker chromosomes (100%) were observed in 2 unexposed individuals (40%). Regarding chtb/chrb, only 2 chtb were observed in two unexposed individuals (40%). About chromosomal fragilities (fra), 4 fragilities were observed in 2 unexposed individuals, all on chromosome 9 [fra (9)(q12)].

CVs and CAs, were also observed in both, exposed residents (R) and unexposed control group (RC) (76 and 29, respectively). However, no statistically significant differences were observed between the group of exposed residents (R) and the unexposed control group (RC) (*p* ≤ 0.3122; unpaired Mann Whitney Wilcoxon test) (Table 2; Figure 1B).

Specifically, in the group of exposed residents, a total of 182 metaphases were analyzed. Among these metaphases, a total of 31 NCAs (40%) were observed in all exposed residents (100%); 6 SCAs were found in 4 (80%) exposed residents; 5 chtb/chrb were observed in 2 (40%) exposed residents, along with 33 fragilities found in all (100%) exposed residents. Additionally, 1 chromosomal heteromorphism (9qh+) was identified (Table 2). Among NCAs, the monosomies (77.4%) were more frequent than trisomies (22.6%). The chromosomes with the highest frequency of monosomies were: chromosome X (16.66%), observed in 3 exposed residents (60%), chromosome 21 (12.5%), observed in 2 (20%) exposed residents, and chromosome 22 (12.4%), observed in 3 exposed residents (30%). Within the trisomies, marker chromosomes were observed more frequently (57%), in 2 exposed residents (40%). Regarding SCAs, a total of 6 SCAs were observed in 4 (80%) exposed residents, with deletions (del) being the most common (50%). Other structural alterations, albeit less frequent, included additions (add) (16.6%), telomeric associations (tas) (16.6%), and isochromosomes (16.6%). The chromosomes involved in SCAs were chromosomes X, 1, 2, 6 and 10. In addition, were observed 5 chtb/chrb, in 2 (40%) exposed residents. Fragilities (fra) were also detected in the exposed residents (33), with fra (9)(q12) being the most prevalent (90.9%), observed in all exposed residents (100%). (Figure 1B; Table 2).

In the unexposed control group (RC), a total of 125 metaphases were analyzed. Among these metaphases, 21 NCAs were observed in all (100%) unexposed individuals, while 6 chrb/chtb were identified in 3 (60%) unexposed individuals. Additionally, 2 fragilities were detected in 1 (20%) unexposed individual. No SCAs or CVs were observed in this group (RC). Monosomies were observed in chromosomes X, 2, 8, 11, 18, 19, and 22, affecting 1 (20%) unexposed individual. Trisomies predominantly involved marker chromosomes (64%), observed in all unexposed individuals (100%). Additionally, chrb/chtb) occurred infrequently, affecting chromosomes X, 1, 2, 12, and 14. Fragilities (fra) were detected in only one unexposed individual (Figure 1B).

### 3.3 NCAs and CVs are associated with exposure time in exposed miners and residents

To ascertain the presence of associations between the frequency of CVs and CAs with variables such as age and coal exposure time (ET) across all groups (M, R, MC, and RC), multivariate analysis was conducted using the Pearson correlation coefficient.

**FIGURE 2:**
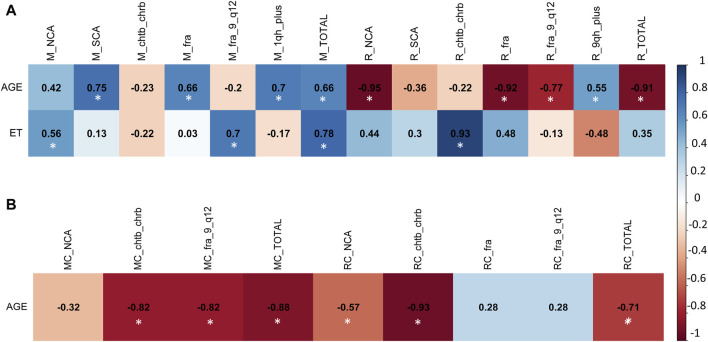
Multivariate analysis with Pearson correlation coefficient for **(A)** Exposed Miners and Exposed Residents, and for **(B)** Miner Control group (unexposed control group) and Resident Control group (unexposed control group). Values greater than 0.5 are indicative of a statistically significant correlation. Abbreviations: M, exposed miners; R, exposed residents; NCAs, numerical chromosomal alterations; SCAs, structural chromosomal alterations; chtb, chromatidic break; chrb, chromosomic break; fra, fragilities; fra (9) (q12), fragility in the long arm of chromosome 9, region 1 and band 2; 1qh+, heterochromatin increased on long arm of chromosome 1; TOTAL, total number of chromosomal variants (CVs) and chromosomal aberrations (CAs).

In the exposed miner group (M), a positive correlation was observed between age and the frequency of SCAs (r = 0.75), fra (r = 0.66), 1qh+ (r = 0.7), and the total alterations present in this group (r = 0.66) (Figure 2A). Additionally, a positive correlation was found between exposure time (ET) and the frequency of NCAs (r = 0.56), fra (9)(q12) (r = 0.7), and the total alterations present in this group (r = 0.78) (Figure 2A). Also, a negative correlation was found between, age with chtb/chrb (r = −0.82), fra (9)(q12) (r = −0.82) and total alterations present in the unexposed group (MC) (r = −0.88) (Figure 2B).

In the resident (R) group, we observed a positive correlation between exposure time (ET) and the frequency of NCAs (r = 0.7), chtb/chrb (r = 0.82), fra (r = 0.65), and the total number of alterations present in this group (Total) (r = 0.79) (Figure 2A). Also, a negative correlation was found between age with NCAs (r = −0.97), chtb/chrb (r = −0.78), fra (r = −0.89) and the total alterations present in the exposed resident (R) group (r = -0.7) (Figure 2A). In addition, in the unexposed resident (RC) group, no linear correlation was found between the frequency of CAs and CVs with age (Figure 2B).

Smoking status was not considered due to its low incidence among individuals. Correlation analyses between drinking status and, CAs and CVs were not conducted because there were no statistically significant differences in this habit (drinking) between the studied groups (M and MC) (*p* > 0.05, unpaired Mann Whitney Wilcoxon test).

### 3.4 Exposed miners and residents exhibit high DNA damage

**FIGURE 3:**
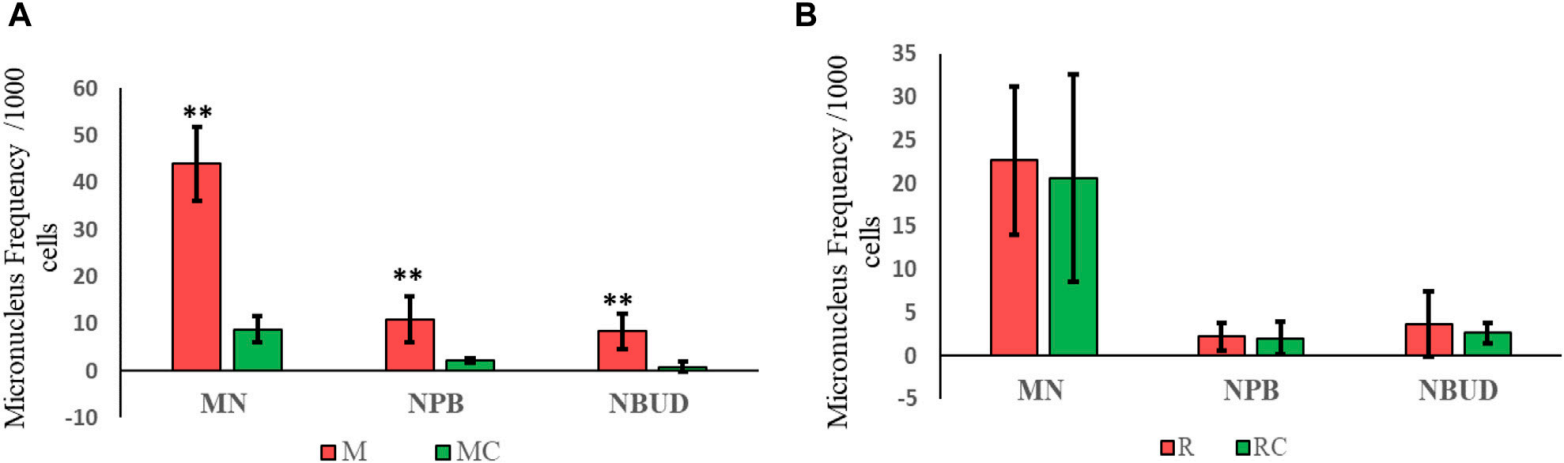
Frequency of micronuclei, nucleoplasmic bridges, and nuclear buds observed in **(A)** Exposed Miners and Miner Control (unexposed control group), and in **(B)** Exposed Residents and Resident Control (unexposed control group). Error bars represents mean standard deviation of 1,000 binucleated cells. **Statistically significant difference relative to unexposed control group at *p* ≤ 0.01 (unpaired Mann Whitney Wilcoxon test). Abbreviations: M, Exposed Miners; MC, Miner control (unexposed control group); R, Exposed Residents; RC, Resident control (unexposed control group); MN, micronuclei; NPB, nucleoplasmic bridges; NBUD, nuclear bud.

**FIGURE 4:**
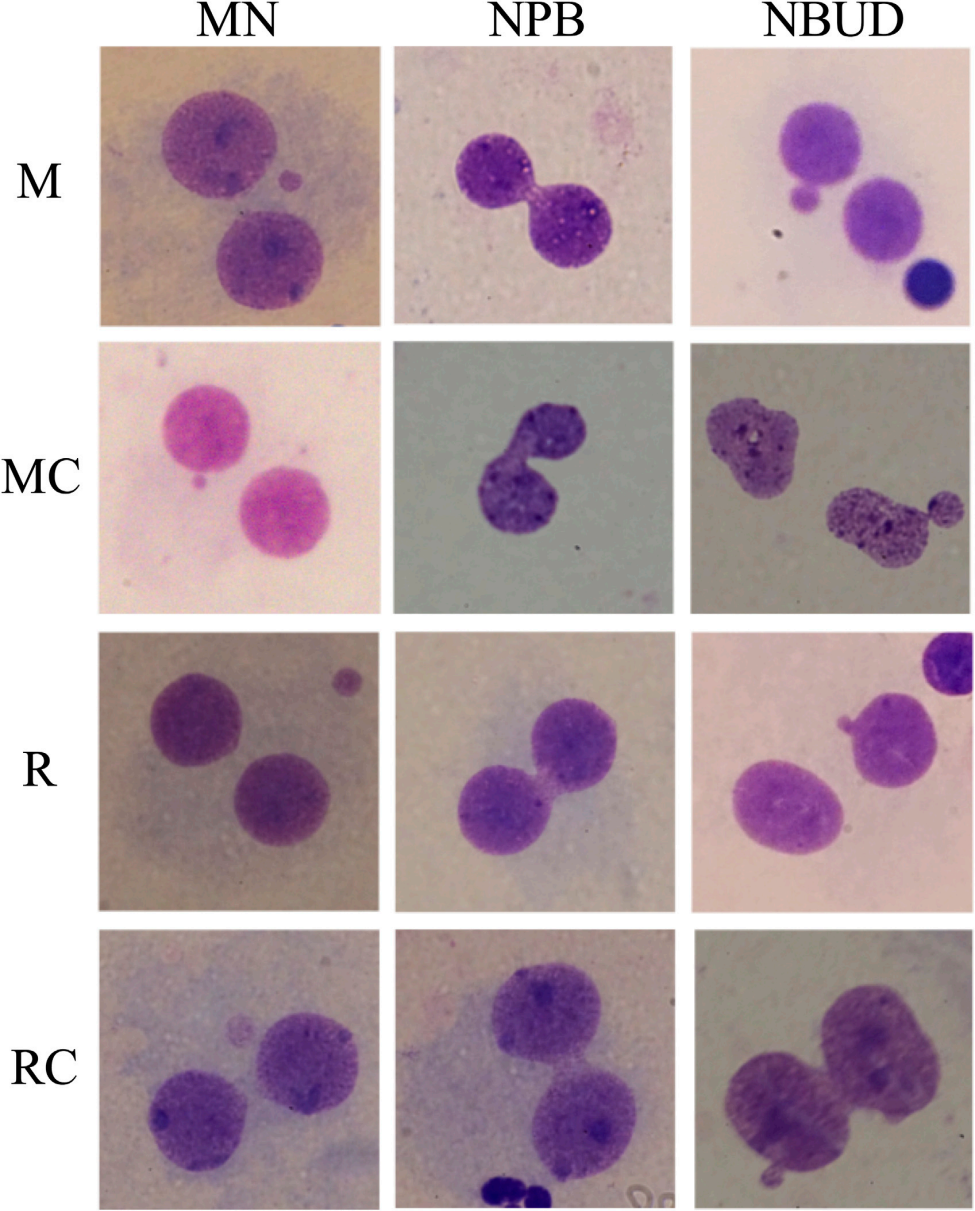
Representative Micronucleus, nucleoplasmic bridges, and nuclear bud images observed in the exposed and unexposed groups. Abbreviations: M, Exposed Miners; MC, Miner control (unexposed control group); R, Exposed Residents; RC, Resident control (unexposed control group); MN, micronuclei; NPB, nucleoplasmic bridges; NBUD, nuclear bud.

A total of 1,000 binucleated cells per individual (both exposed miners and exposed residents, as well as their respective controls) were examined to assess the presence of MN, NPB, and NBUD. In total, 20,000 binucleated cells were analyzed (Figures 3, 4).

In exposed miners, statistically significant differences (*p* ≤ 0.01) were observed in the frequency of MN, NPB and NBUD (44 ± 7.8; 11 ± 4.8 and 8.4 ± 3.8, respectively), compared to the frequencies observed in the unexposed control group (8.8 ± 2.8; 2.2 ± 0.5 and 0.8 ± 1.1, respectively) (Figures 3A, 4).

In exposed residents, no statistically significant differences (*p* > 0.05, Student’s t-test) were observed in the frequency of MN, NPB and NBUD (22.6 ± 8.6; 2.2 ± 1.6 and 3.6 ± 3.8, respectively), compared to the frequencies observed in the unexposed control group (20.6 ± 12; 2 ± 1.9 and 2.6 ± 1.1, respectively) (Figures 3B, 4).

### 3.5 Nuclear buds are associated with exposure time in exposed miners and residents

To investigate associations between the frequency of MN, NPB, and NBUD and variables such as age and coal exposure time (ET) across all groups [exposed miners (M and R) and unexposed control groups (MC and RC)], multivariate analysis using the Pearson correlation coefficient was conducted. Smoking status was not considered due to its low incidence among individuals.

**FIGURE 5:**
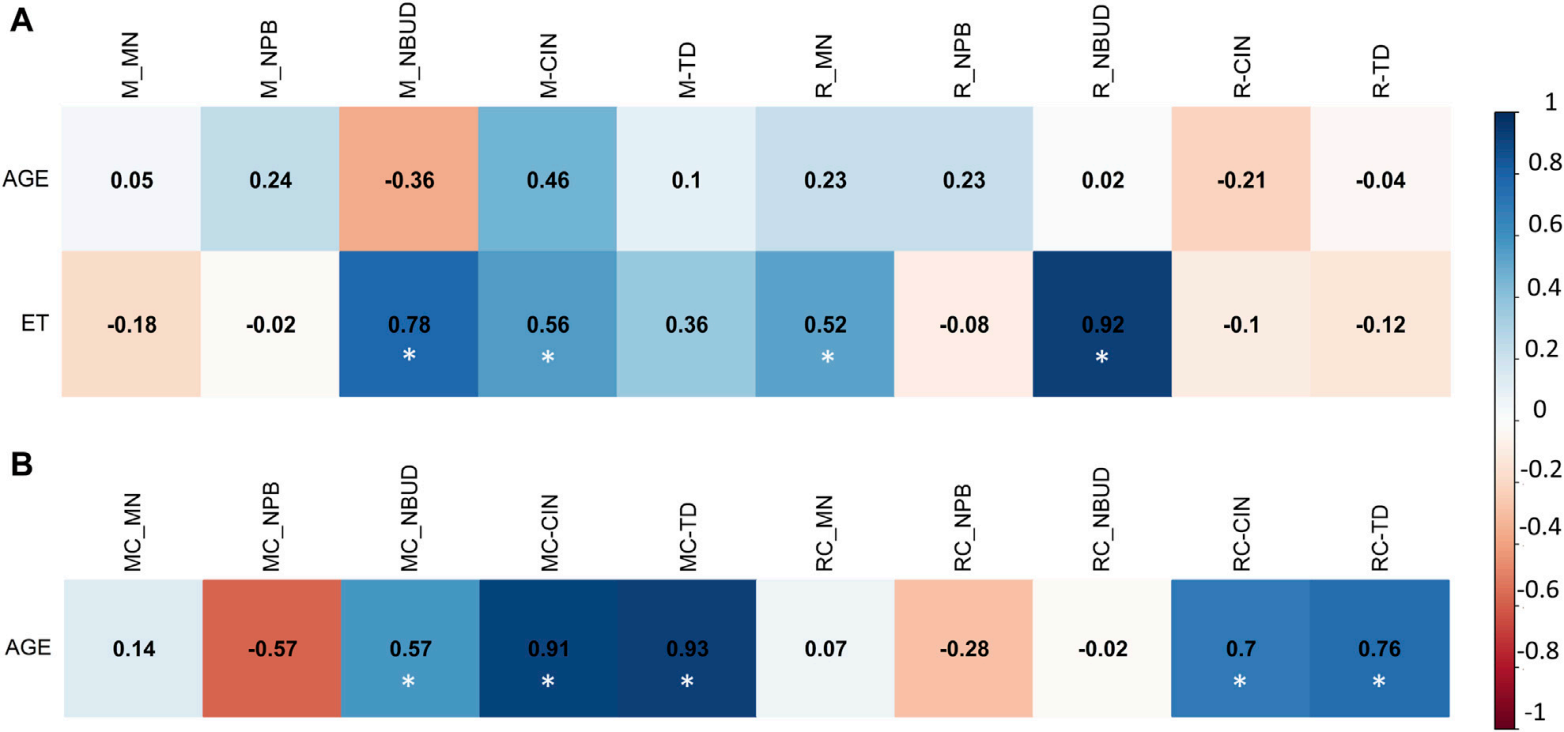
Multivariate analysis with Pearson correlation coefficient for **(A)** Exposed Miners and Exposed Residents, and for **(B)** Miner Control group (unexposed control group) and Resident Control group (unexposed control group). Values greater than 0.5 are indicative of a statistically significant correlation. Abbreviations: M, exposed miners; R, exposed residents; MN, Micronucleus; NPB, nucleoplasmic bridge; NBUD, nuclear buds; CIN, Chromosomal Instability; TD, True Diversity index; ET, coal exposure time.

In the exposed miner group (M), a strong positive correlation was found between ET and the frequency of NBUD (r = 0.78) (Figure 5A). However, while in the exposed miner group (M) no linear correlation was found between age and MN, NPB, and NBDU, in the unexposed miner control group (MC) a positive correlation was observed between age and NBUD (r = 0.59) (Figure 5B).

Similar to the findings in the exposed miner group (M), the exposed resident group (R) also exhibited a strong positive correlation between ET and the frequency of NBUD (r = 0.92) (Figure 5A). However, in the resident control group (RC), no linear correlation was observed between age and MN, NPB, and NBDU (Figure 5B). Correlation analyses regarding drinking status and MN, NPB, and NBUD, were not conducted due to the lack of statistically significant differences in drinking habits between the studied groups (M and MC) (*p* > 0.05, unpaired Mann Whitney Wilcoxon test).

### 3.6 Exposed miners and residents exhibit high levels of CIN and CH

**FIGURE 6:**
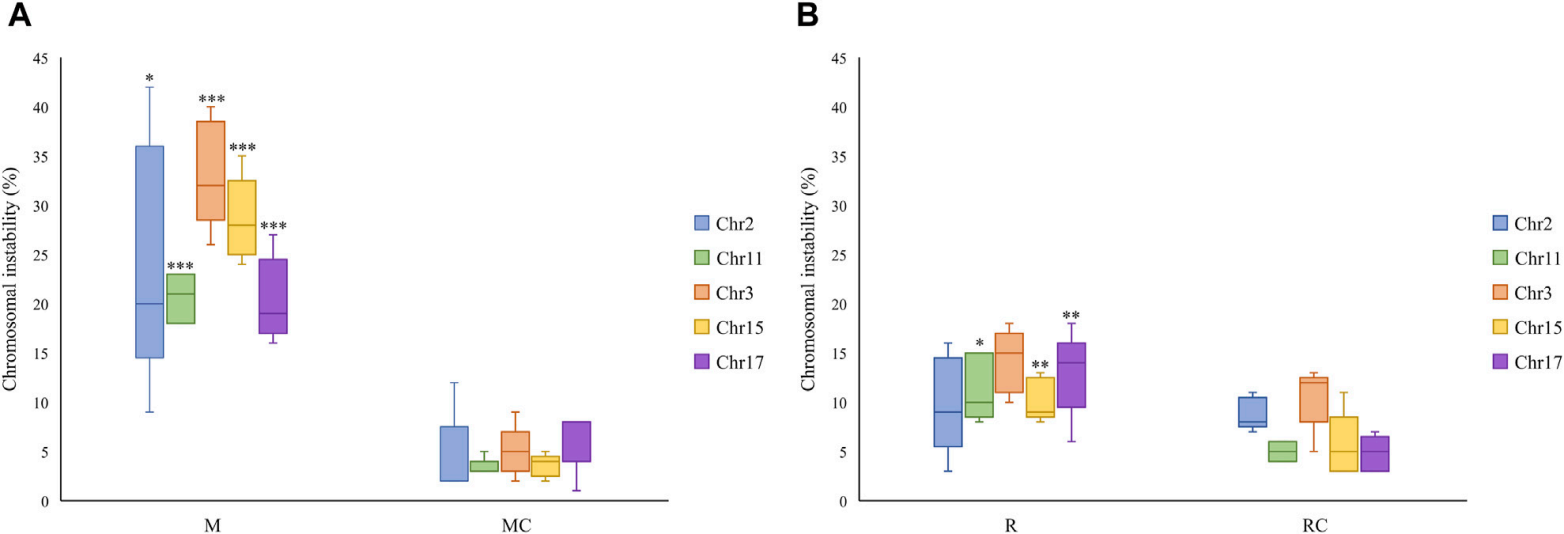
Chromosomal instability observed in **(A)** Exposed Miners and Miner Control group and **(B)** Exposed Residents and Resident Control group. Error bars represents mean standard deviation for 100 interphase cells. *Statistically significant difference relative to unexposed control group at *p* ≤ 0.05 (Students’ t-test). **Statistically significant difference relative to unexposed control group at *p* ≤ 0.01. ***Statistically significant difference relative to unexposed control group at *p* ≤ 0.001 (Student’s t-test). Abbreviations: M, Exposed Miners; MC, Miner control (unexposed control group); R, Exposed Residents; RC, Resident control (unexposed control group); Chromosomal instability (CIN).

**FIGURE 7:**
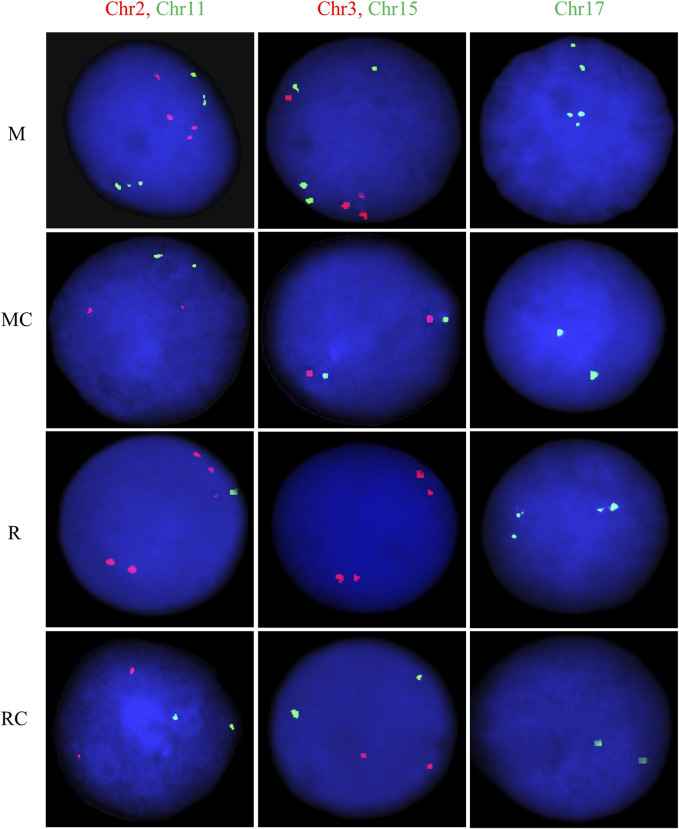
Representative FISH images for exposed and unexposed groups. Dual-color FISH was performed on nuclei spreads for chromosomes 2 (CEP2; *Spectrum Orange*) and 11 (CEP11; *Spectrum Green*), as well as, for chromosomes 3 (CEP3; *Spectrum Orange*) and 15 (CEP15; *Spectrum Green*). For chromosome 17 (CEP17; *Spectrum Green*), single FISH was performed. One hundred cells per chromosome were counted for each individual. Abbreviations: M, Exposed Miners; MC, Miner control (unexposed control group); R, Exposed Residents; RC, Resident control (unexposed control group).

The exposed miners displayed significantly higher CIN levels (25% ± 2) compared to the unexposed control group, which showed lower CIN (5% ± 1) (Figures 6A, 7; Supplementary Table S2). This difference in CIN levels between exposed miners and the control group was statistically significant (*p* ≤ 0.001; Student’s t-test).

In the exposed resident group, it is noteworthy that the level of CIN was significantly higher (*p* ≤ 0.01**; Student’s t-test) compared to the control group (12 ± 2.3 and 7 ± 1.5, respectively) (Figures 6B, 7; Supplementary Table S2). However, the CIN level observed in the exposed resident group was lower than this observed in the exposed miner group.

Regarding the chromosomes with the highest and lowest levels of CIN, opposite trends were observed between the exposed miners and the unexposed group. Specifically, while in exposed miners (M), chromosome 17 displayed the lowest CIN level (20% ± 4), in the unexposed group (MC), chromosome 17 showed the highest CIN level (6% ± 3) (Supplementary Table S2). In contrast to the findings in exposed miners, both the exposed residents (R) and the control group (RC), showed that chromosome 3 had the highest CIN levels (14% ± 3.19% and 11% ± 3.2, respectively) (Supplementary Table S2).

Regarding clonal heterogeneity (CH), exposed miners demonstrated high CH levels (2.25 ± 0.13), whereas the unexposed group exhibited low CH levels (1.23 ± 0.05) (Supplementary Table S2; Supplementary Figure S1). These differences were statistically significant (*p* < 0.001***; Non-parametric Mann-Whitney Wilcoxon test). Similar results were observed in the exposed resident group, where intermediate levels of CH (1.56 ± 0.09) were evident compared to low CH levels (1.33 ± 0.06) observed in the control group (Supplementary Table S2; Supplementary Figure S1). These differences were also statistically significant (*p* < 0.01**; Non-parametric Mann-Whitney Wilcoxon test).

### 3.7 CIN and CH are associated with exposure time in exposed miners

To investigate associations between CIN and CH with variables such as age and coal exposure time (ET) across all groups [exposed miners (M and R) and unexposed (MC and RC)], multivariate analysis using the Pearson correlation coefficient was conducted.

**FIGURE 8:**
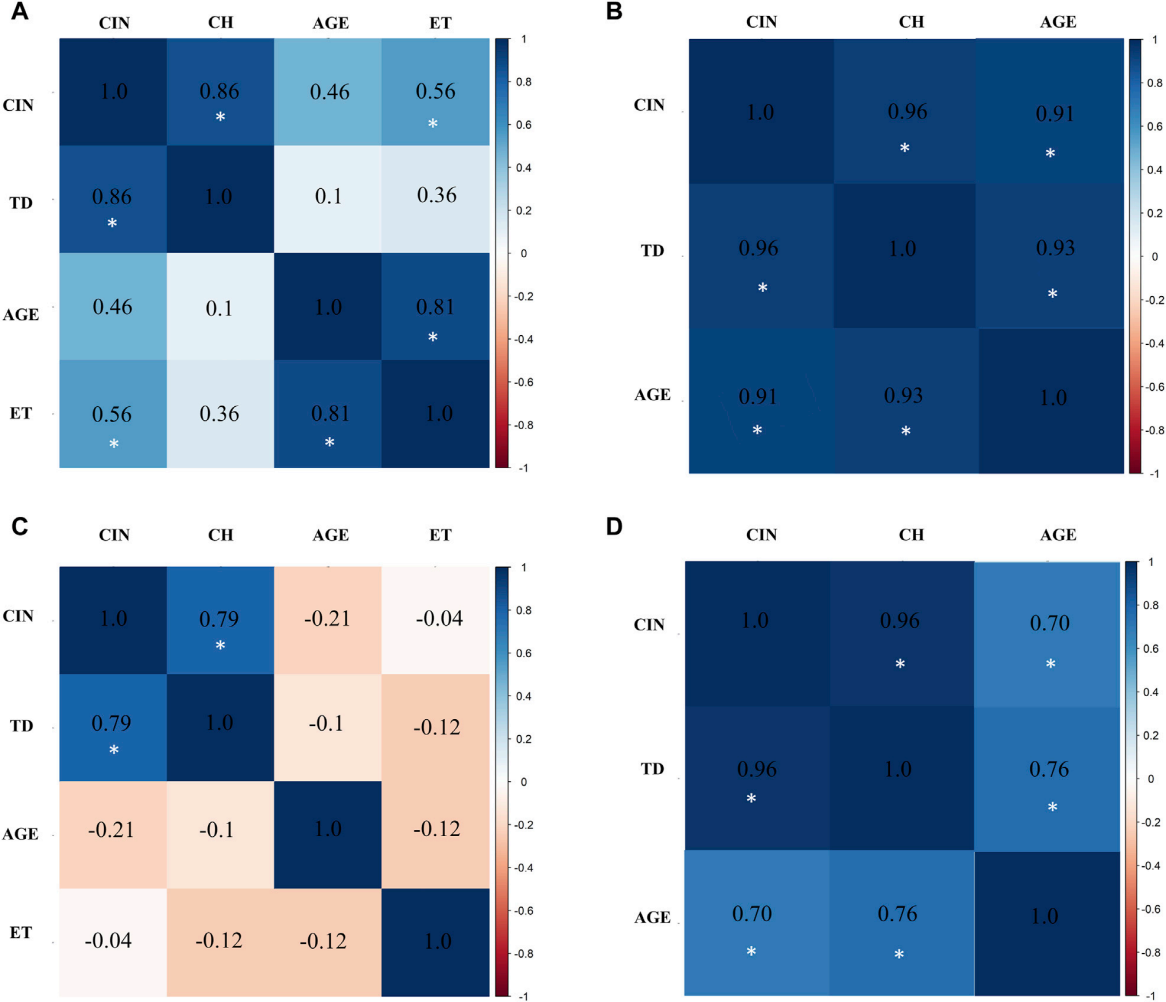
Multivariate analysis with Pearson correlation coefficient for **(A)** Exposed Miners, **(B)** Miner Control (unexposed control group), **(C)** Exposed Residents, and **(D)** Resident Control (unexposed control group). Values greater than 0.5 are indicative of a statistically significant correlation. Abbreviations: CIN, Chromosomal Instability; TD, True Diversity; ET, coal exposure time.

In the exposed miner group (M), a significant positive correlations were observed between: chromosomal instability (CIN) and true diversity index (TD) (r = 0.8636211), between age and chromosomal instability (CIN) (r = 0.4607530), and between exposure time (ET) and chromosomal instability (CIN) (r = 0.5586379) (Figure 8A). In the control miner group (MC), significant positive correlations were observed between chromosomal instability (CIN) and age (r = 0.9136567), and between clonal heterogeneity (CH) and age (r = 0.9264975) (Figure 8B).

Similar to what was observed in the exposed miners, in the exposed residents a strong positive correlation between chromosomal instability (CIN) and true diversity index (TD) (r = 0.7915529) was also found. However, negative correlations were observed between age and chromosomal instability (CIN) (r = −0.2139315), and between exposure time (ET) and true diversity index (TD) (Figure 8C). In the control resident group (RC), a significant positive correlation between age and chromosomal instability (CIN) (r = 0.6957846), and between clonal heterogeneity (CH) and age (r = 0.7581412) were also observed (Figure 8D). It is important to highlight that in both exposed groups (M and R), age was not directly correlated with the duration of exposure. Older individuals did not necessarily have the longest exposure times (Supplementary Table S1).

### 3.8 Chromosomal alterations (CAs) are associated with age and with exposure time in exposed miners

To confirm the effects of occupational exposure, a multiple regression analysis (multiple linear regression model) was conducted. In the exposed miner group (M), a significant positive correlation (0.690519) was observed between the total number of CAs and age (AGE) (*p* = 0.00573**), indicating that for each additional year in a miner’s age, the number of CAs increases by 0.69 units (Supplementary Figure S2A). A similar trend was observed when comparing the total number of CAs with exposure time (ET), where a significant (*p* = 0.00388**) positive correlation (0.057312) was also observed (Supplementary Figure S2B). This means that for each additional unit of ET, there is an increase of 0.057312 in the number of CAs. In the control miner group (MC), a significant (*p* = 0.0499*) negative correlation (−0.28226) was observed between the number of CAs and age (AGE) (Supplementary Figure S2C). In the exposed resident group (R), no significant correlations were observed (Supplementary Figure S2D). No other significant correlations were found across all groups [exposed miners (M and R) and unexposed (MC and RC)].

## 4 Discussion

Coal miners are constantly exposed to coal dust and its derivatives in the workplace, especially during underground mining. This particulate material, when inhaled can be deposited in the lungs, altering the parameters of oxidative damage (Pinho et al., 2004), and causing the development of different diseases (Donbak et al., 2005). In Colombia, coal extraction is carried out both, open pit and underground, and although the human health problems represented by exposure to coal in open pit mining workers are known, studies that report chromosomal and genotoxic damage in underground coal miners are scarce or absent. Our findings reveal a statistically significant increase in chromosomal aberrations (NCAs, SCAs, CIN, and CH) and genotoxic damage (MN, NPB, and NBDU) among underground coal miners compared to the low frequency observed in the unexposed control group. In fact, the average number of CVs and CAs observed in underground coal miners was fifty times greater than that seen in the unexposed individuals. These results suggest a potential cytogenetic impact of coal exposure on underground mining workers. Indeed, underground coal mining poses a heightened risk for miners, attributed not only to the threat of methane and coal dust explosions (James R and Raji, 2001), but also to continuous exposure to coal dust and its byproducts in the work environment.

Furthermore, a statistically significant increase in the frequency of chrb and chtb, was noted in the underground coal miners’ group compared to the low frequency observed in the unexposed control group. These aberrations entail single and/or double-stranded DNA breaks (DSBs), with the latter being particularly concerning. DSBs can occur when lesions, such as those initiated by DNA oxidation, are not properly repaired. Reactive oxygen species (ROS) can cause DNA damage, leading to oxidative lesions. If this damage is not correctly repaired, it can be potentially devastating to normal cell physiology, resulting in mutagenesis and/or cell death. Indeed, damage-induced mutagenesis has been linked to various malignancies (Maynard et al., 2008; Varona et al., 2018).

The identification of chrb and chtb in underground coal miners, suggests a heightened susceptibility to develop complex chromosomal rearrangements, such as translocations, inversions, dicentric chromosomes, deletions, and duplications, among others. These findings underscore the substantial chromosomal damage linked to coal exposure observed in our study.

**FIGURE 9:**
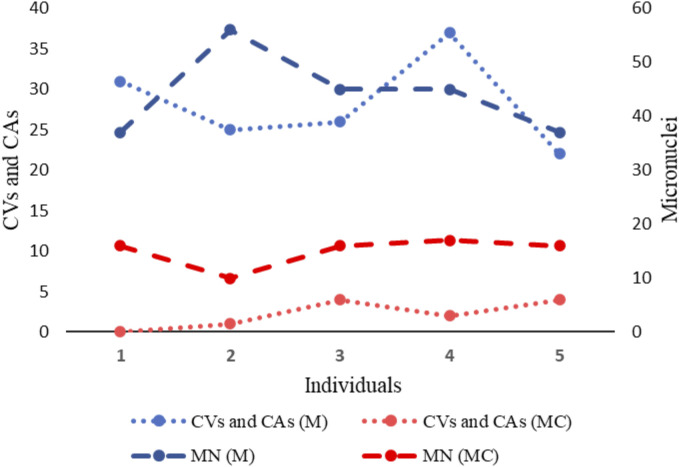
Two-axis graph representing the frequency of Chromosomal Variants (CVs), Chromosomal Aberrations (CAs) and Micronuclei (MN) in exposed miners (M) and in unexposed control group (MC). On the left axis of the graph, the mean frequency of CVs and CAs is indicated. On the right axis of the graph, the mean frequency of MN is indicated.

Regarding the evaluation of genotoxic damage, we found that the underground coal miners, presented a statistically significant high frequency of MN, NPB and NBUD, compared to what was observed in the unexposed control group. In fact, while the frequency of MN observed in underground coal miners, was almost forty times higher than that observed in unexposed individuals, the frequency of NPB and NBUD observed in underground coal miners, was approximately eight times higher than that observed in unexposed individuals. It is well-established that MN can arise due to deficiencies in cellular repair mechanisms and the accumulation of DNA damage and chromosomal aberrations, indicating a clastogenic influence on the organism. Therefore, the elevated frequency of chromosomal aberrations detected in underground coal miners could reflect the genetic damage evidenced by the presence of MN. Indeed, our findings not only show correlations between the frequency of MNs and chromosomal aberrations, but also underscore the substantial genetic damage present in underground coal miners (Figure 9).

Our results are consistent with the few previous studies carried out in this regard, where it was reported that underground coal miners presented a significant increase in genetic damage (CAs and MN) compared to the control group (Maley et al., 2006; Sinitsky et al., 2016). Actually, MN have become the most prevalent biomarker of chromosomal defects induced by genotoxic agents (Luzhna et al., 2013). For instance, it has been indicated that the presence of MN may predispose cells to present both NCAs and SCAs (Gisselsson et al., 2001; Fenech, et al., 2011). Mechanisms associated with MN formation encompass the faulty repair of double-stranded DNA breaks (DSBs), which may result in both symmetrical and asymmetrical chromatid and chromosome exchanges, along with chromatid and chromosome fragments (Sinitsky et al., 2016). Furthermore, it has been indicated that during anaphase, rearranged chromosomes (chromosomes with structural alterations) often fail to segregate in an orderly manner, leading to the formation of nucleoplasmic bridges (NPB) between the spindle poles (Caradonna, 2015). As result of the formation of NPB, the lagging chromosome may be lost, form a MN, or be randomly incorporated into either of the daughter nuclei, conducing to NCAs or aneuploidy. Moreover, at the anaphase-telophase transition, these NPB may subsequently break, resulting in novel SCAs in the daughter cells (Rehn et al., 2003; Caradonna, 2015). It is important to note that these abnormal nuclear shapes are recognized as common characteristics observed in a broad spectrum of chromosomally unstable cells (Caradonna, 2015; Volobaev et al., 2018), and also as biomarkers of DNA misrepair and/or telomere end-fusions (Sinitsky et al., 2016).

Regarding exposed residents (R), although no significant differences were observed in the frequency of chromosomal aberrations and genotoxic damage compared to the resident control group (RC), RC individuals showed more chromosomal alterations than MC individuals. However, these differences were not statistically significant. Differences in both control groups (MC and RC) were represented by the higher frequency of trisomies in only one of the resident control individuals (RC3), compared to no trisomy observed in the respective exposed resident (R3). The variations in the frequency of chromosomal alterations among normal individuals of the same age and gender, who are not exposed to genotoxic agents, can be attributed to several factors, including: genetic diversity (each individual has a unique genetic makeup, which can influence the stability of their chromosomes) (Jordan, 2021); natural cellular processes (errors during DNA replication, mitosis, or meiosis can lead to chromosomal alterations) (Potapova and Gorbsky, 2017); epigenetic factors (DNA methylation and histone modification, can affect gene expression and chromosomal stability) (Li et al., 2021), and random chance (random errors during cell division and DNA replication can lead to chromosomal alterations, and these events can vary from person to person simply due to chance) (Acuna-Hidalgo et al., 2016). These factors, individually or in combination, could contribute to the observed variations in chromosomal alterations among individuals who are otherwise considered healthy and not exposed to specific genotoxic agents.

The findings of CIN evaluation by FISH analysis, suggest a possible direct link between coal dust exposure and chromosomal instability. Although the exact mechanisms underlying the induction of chromosomal (chromosomal aberrations, CIN and CH) and genotoxic damage by coal, remain incompletely understood, it has been proposed that coal exposure triggers heightened production of ROS. ROS, induce fragmentation and oxidation of nucleic acids, proteins, and lipids (Kaur and Kaur, 2018). Oxidative damage to DNA caused by ROS, leads to the formation of lesions such as 8-oxoguanine (8-oxoG) and abasic (AP) sites. If these lesions are not repaired before DNA replication, they can cause replication forks to collapse, resulting in DSBs. Inaccurate repair of these DSBs through mechanisms like homologous recombination (HR), and non-homologous end joining (NHEJ) can induce numerical and structural chromosomal aberrations (Korolev, 2005), contributing to genomic instability and the potential development of cancer and other diseases. When a DSB occurs, the ends are highly recombinogenic and can invade DNA duplexes at sites with sequence homology. This invasion initiates homologous recombination repair (HRR), which can lead to crossover events in the double-strand break repair (DSBR). HRR has a significant potential to induce exchange-type chromosomal alterations, such as translocations, inversions, and deletions, especially if the invasion occurs at repeat sequences on different chromosomes (ectopic recombination). Even a single DSB can trigger these aberrations if ectopic recombination happens between repeated sequences located on the same or different chromosomes (Pfeiffer et al., 2000). Indeed, it has been suggested that ROS may promote inflammatory processes, thereby modulating the overall extent of cytogenetic damage in miners (Duval et al., 2003; Kopprasch et al., 2003; Volobaev et al., 2016), as observed in our study. In fact, the inflammatory process associated with some genotoxic responses has been extensively documented (Rehn et al., 2003; Karlsson et al., 2006; Jacobsen et al., 2009).

One of the structural chromosomal aberrations observed altered in our study, in more of one underground coal miners, was del (18)(p11). This affected chromosomal region have been reported in various types of cancer, including acute lymphoblastic leukemia/lymphoblastic lymphoma (Udayakumar et al., 2007), uterine leiomyomata (Christacos et al., 2006), synovial sarcoma (Iliszko et al., 2009; Przybyl et al., 2012), among others. In fact, the International Agency for Research on Cancer, indicated that people who are exposed to coal dust, have an increased risk of cancer, especially lung and gastric cancer (Swaen et al., 1995).

Likewise, we noted statistically significant high levels of clonal heterogeneity in both the exposed group (miners and residents) compared to the low levels observed in their respective control groups. High clonal heterogeneity in exposed coal miners, could have significant implications. For example, this variability within the cellular population could indicate increased genomic instability and a greater likelihood of acquiring mutations or genetic alterations over time. Consequently, individuals with high clonal heterogeneity may face elevated risks of developing cancer or other diseases associated with genetic instability. Overall, the increased clonal heterogeneity observed in exposed coal miners underscores the importance of comprehensive surveillance and intervention strategies to mitigate the adverse health effects associated with occupational coal exposure.

Notably, our study revealed a positive correlation between, age and exposure time (ET) with chromosomal instability (CIN) and CAs in exposed miners. Age and duration of coal exposure are factors that may contribute to high levels of chromosomal instability. These results suggested that as individuals age, their cells become more prone to genetic alterations. Additionally, prolonged exposure to coal dust and its derivatives, has been associated with increased oxidative stress and DNA damage, which can further exacerbate chromosomal instability. This combination of factors may lead to a higher frequency of chromosomal aberrations and genetic damage in individuals exposed to coal over extended periods, potentially increasing their susceptibility to diseases associated with genomic instability.

Overall, our results, in addition to reflecting genotoxic damage in the exposed population, show an increased risk of DNA and chromosome damage in underground coal miners. Given the pivotal role of chromosomal aberrations, chromosomal instability, and clonal heterogeneity in cells as quantitative parameters of mutagenesis (Volobaev et al., 2016), the evaluation of chromosomal and genotoxic damage can offer valuable insights into the effects induced by genotoxic agents in coal miners.

The limitation of our study was the restricted access to the samples, related to people’s refusal to participate. Despite this constraint, the comprehensive analysis of between 11 and 45 metaphases, and 1,000 binucleated cells, per individual, ensures the robustness and accuracy of our results. Our pilot study suggests that occupational exposure to coal induces chromosomal damage in underground coal miners, highlighting the importance of validating these findings with a larger sample size.

## 5 Conclusion

The results of our study, despite being conducted on a limited sample size, suggest that occupational exposure to coal from underground mining, induces chromosomal and genotoxic damage. Chromosomal damage is an important step in carcinogenesis and the development of many other diseases. The findings provide valuable insights into the effects of coal dust exposure on chromosomal integrity and genetic stability. Furthermore, this research underscores the urgency of implementing preventive measures and occupational safety protocols to safeguard the health and wellbeing of coal miners.

## Data Availability

The original contributions presented in the study are included in the article/Supplementary Material, further inquiries can be directed to the corresponding authors.
