# Supplementary material for: Unraveling chromosomal and genotoxic damage in individuals occupationally exposed to coal from underground mining
Source: Front Genet. 2024 Jul 4;15:1422938. doi: 10.3389/fgene.2024.1422938 (PMC11254797; doi:10.3389/fgene.2024.1422938)
Supplement: Supplementary file 1 [file Table1.DOCX]

Supplementary Material

# Supplementary Table S1. Detailed characteristics of the groups studied. A) Exposed Miners and unexposed control. B) Exposed Residents and unexposed group

| **A** | **M** | **Age (y)** | **Exposure (m)** | **Habits** | | **MC** | **Age (y)** | **Exposure (m)** | **Habits** | |
| --- | --- | --- | --- | --- | --- | --- | --- | --- | --- | --- |
|  |  |  |  | **S** | **D** |  |  |  | **S** | **D** |
|  | 1 | 52 | 180 | - | - | 1 | 53 | - | - | 1 / m |
|  | 2 | 47 | 120 | - | 1 / y | 2 | 44 | - | - | 1 / w |
|  | 3 | 40 | 228 | - | - | 3 | 40 | - | - | 1 / m |
|  | 4 | 48 | 324 | - | 1 / w | 4 | 48 | - | - | 1 / m |
|  | 5 | 40 | 168 | - | 1 / y | 5 | 40 | - | - | 1 / m |
|  | **Mean** | 45.4 | 204 |  | | **Mean** | 45 |  | | |
|  | **Median** | 47 | 180 |  |  | **Median** | 44 |  |  |  |
|  | **SD** | 5.3 | 77.3 |  |  | **SD** | 5.6 |  |  |  |
|  |  |  |  |  |  |  |  |  |  |  |
| **B** | **R** | **Age (y)** | **Exposure (m)** | **Habits** | | **RC** | **Age (y)** | **Exposure (m)** | **Habits** | |
|  |  |  |  | **S** | **D** |  |  |  | **S** | **D** |
|  | 1 | 48 | 576 | - | 1 / w | 1 | 48 | - | - | 1 / y |
|  | 2 | 25 | 300 | - | - | 2 | 23 | - | - | - |
|  | 3 | 55 | 300 | - | - | 3 | 54 | - | - | - |
|  | 4 | 49 | 132 | - | 1 / y | 4 | 49 | - | - | 1 / m |
|  | 5 | 61 | 132 | - | - | 5 | 60 | - | - | - |
|  | **Mean** | 47,6 | 288 |  | | **Mean** | 46,8 |  | | |
|  | **Median** | 49 | 300 |  |  | **Median** | 49 |  |  |  |
|  | **SD** | 13.7 | 181.6 |  |  | **SD** | 14.1 |  |  |  |

**Abbreviations:** M, Exposed Miner; MC, Miner control (Unexposed group); R, Exposed Resident; RC, Resident control (Unexposed group); S, Smoking; D, Drinking; m, month; w, week; y, year; SD, Standard Deviation.

**Supplementary Table S2.** Chromosomal Instability (CIN) and True Diversity Index (TD) in Exposed and Unexposed groups

| **M** | **CIN** | | | | | | **TD** | | | | | |
| --- | --- | --- | --- | --- | --- | --- | --- | --- | --- | --- | --- | --- |
|  | **CEP2** | **CEP3** | **CEP11** | **CEP15** | **CEP17** | **TOTAL** | **CEP2** | **CEP3** | **CEP11** | **CEP15** | **CEP17** | **TOTAL** |
| 1 | 42 | 37 | 23 | 26 | 18 | **29** | 2.971 | 2.919 | 2.074 | 2.277 | 1.930 | **2.434** |
| 2 | 20 | 40 | 18 | 30 | 19 | **25** | 1.785 | 2.575 | 1.846 | 2.622 | 1.945 | **2.154** |
| 3 | 20 | 31 | 18 | 28 | 27 | **25** | 1.936 | 2.743 | 1.763 | 2.611 | 2.287 | **2.268** |
| 4 | 9 | 26 | 21 | 35 | 22 | **23** | 1.440 | 2.206 | 2.102 | 2.435 | 2.331 | **2.103** |
| 5 | 30 | 32 | 23 | 24 | 16 | **25** | 2640 | 2.801 | 2.098 | 2.195 | 1.806 | **2.308** |
| Mean | 24 | 33 | 21 | 29 | 20 | **25** | 2.154 | 2.649 | 1.976 | 2.428 | 2.060 | **2.253** |
| SD | 12 | 5 | 3 | 4 | 4 | **2** | 0.632 | 0.2767 | 0.1602 | 0.1923 | 0.234 | **0.1308** |
| **MC** | **CIN** | | | | | | **TD** | | | | | |
|  | **CEP2** | **CEP3** | **CEP11** | **CEP15** | **CEP17** | **MEAN** | **CEP2** | **CEP3** | **CEP11** | **CEP15** | **CEP17** | **MEAN** |
| 1 | 12 | 9 | 5 | 3 | 1 | **6** | 1.611 | 1.472 | 1.279 | 1.166 | 1.058 | **1.317** |
| 2 | 3 | 2 | 3 | 5 | 7 | **4** | 1.144 | 1.103 | 1.344 | 1.220 | 1.289 | **1.220** |
| 3 | 2 | 5 | 3 | 4 | 8 | **4** | 1.103 | 1.250 | 1.144 | 1.210 | 1.322 | **1.206** |
| 4 | 2 | 5 | 3 | 4 | 8 | **4** | 1.113 | 1.250 | 1.244 | 1.210 | 1.322 | **1.228** |
| 5 | 2 | 4 | 3 | 2 | 8 | **4** | 1.105 | 1.210 | 1.164 | 1.103 | 1.322 | **1.181** |
| Mean | 4 | 5 | 3 | 4 | 6 | **5** | 1.215 | 1.257 | 1.235 | 1.182 | 1.262 | **1.230** |
| SD | 4 | 3 | 1 | 1 | 3 | **1** | 0.2221 | 0.1345 | 0.0825 | 0.0486 | 0.1152 | **0.0518** |
| **R** | **CIN** | | | | | | **TD** | | | | | |
|  | **CEP2** | **CEP3** | **CEP11** | **CEP15** | **CEP17** | **MEAN** | **CEP2** | **CEP3** | **CEP11** | **CEP15** | **CEP17** | **MEAN** |
| 1 | 13 | 12 | 8 | 9 | 6 | **10** | 1.711 | 1.712 | 1.440 | 1.522 | 1.333 | **1.544** |
| 2 | 9 | 18 | 15 | 13 | 14 | **14** | 1.845 | 1.567 | 1.455 | 1.498 | 1.389 | **1.551** |
| 3 | 16 | 16 | 15 | 9 | 14 | **14** | 1.816 | 1.722 | 1.832 | 1.440 | 1.610 | **1.684** |
| 4 | 8 | 15 | 9 | 12 | 18 | **12** | 1.382 | 1.845 | 1.433 | 1.574 | 1.827 | **1.612** |
| 5 | 3 | 10 | 10 | 8 | 13 | **9** | 1.166 | 1.430 | 1.500 | 1.382 | 1.692 | **1.434** |
| Mean | 10 | 14 | 11 | 10 | 13 | **12** | 1.584 | 1.655 | 1.532 | 1.483 | 1.570 | **1.565** |
| SD | 4.9699 | 3.1937 | 3.3615 | 2.1679 | 4.3589 | **2.3983** | 0.2971 | 0.1598 | 0.1699 | 0.0742 | 0.2069 | **0.0925** |
| **RC** | **CIN** | | | | | | **TD** | | | | | |
|  | **CEP2** | **CEP3** | **CEP11** | **CEP15** | **CEP17** | **MEAN** | **CEP2** | **CEP3** | **CEP11** | **CEP15** | **CEP17** | **MEAN** |
| 1 | 8 | 5 | 4 | 3 | 3 | **5** | 1.362 | 1.261 | 1.210 | 1.166 | 1.144 | **1.229** |
| 2 | 7 | 12 | 6 | 3 | 3 | **6** | 1.378 | 1.602 | 1.255 | 1.167 | 1.145 | **1.309** |
| 3 | 8 | 13 | 4 | 11 | 5 | **8** | 1.362 | 1.678 | 1.212 | 1.537 | 1.220 | **1.402** |
| 4 | 11 | 12 | 6 | 6 | 7 | **8** | 1.462 | 1.558 | 1.308 | 1.304 | 1.289 | **1.384** |
| 5 | 10 | 11 | 5 | 5 | 6 | **7** | 1.430 | 1.520 | 1.261 | 1.261 | 1.255 | **1.345** |
| Mean | 9 | 11 | 5 | 6 | 5 | **7** | 1.399 | 1.524 | 1.249 | 1.287 | 1.211 | **1.334** |
| SD | 1.6432 | 3.2094 | 1 | 3.2863 | 1.7889 | **1.5773** | 0.0452 | 0.1581 | 0.0406 | 0.1521 | 0.0648 | **0.0688** |

**Abbreviations:** M, Exposed Miner; MC, Miner control (Unexposed group); R, Exposed Resident; RC, Resident control (Unexposed group); CEP2, centromeric probe for chromosome 2; CEP3, centromeric probe for chromosome 3; CEP11, centromeric probe for chromosome 11; CEP15, centromeric probe for chromosome 15; CEP17, centromeric probe for chromosome 17; CIN, Chromosomal Instability; TD, True Diversity index; SD, Standard Deviation

##

**Supplementary Figure S1.** Clonal heterogeneity determined by True Diversity index for (A) Exposed Miners and unexposed group and (B) Exposed Resident and unexposed group. Abbreviations: CH, Clonal heterogeneity; M, Exposed Miners; MC, Miner control (unexposed control group); R, Exposed Residents; RC, Resident control (unexposed control group).


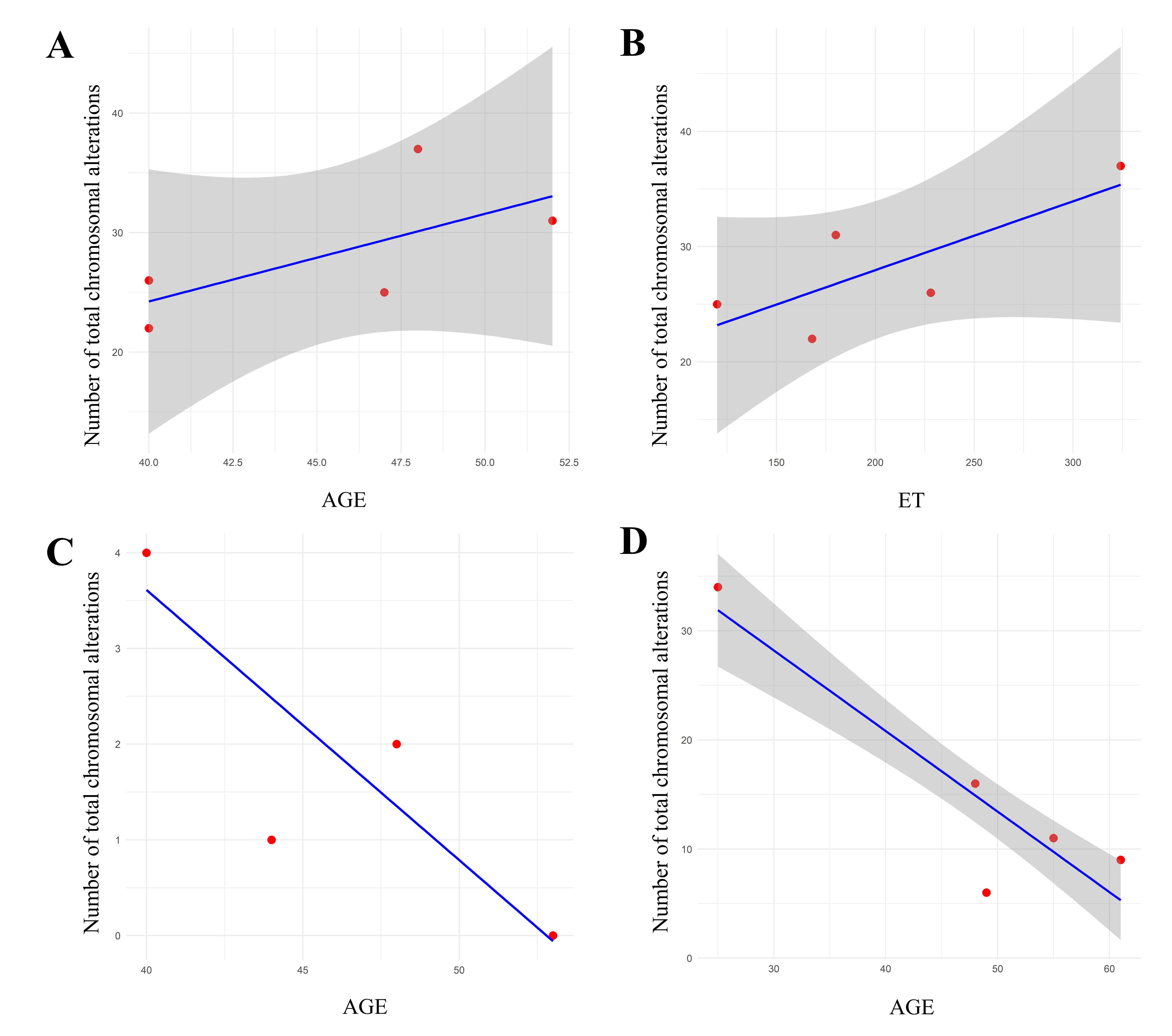


**Supplementary Figure S2.** Multivariate regression analysis for the comparison of association between chromosomal alterations, age and exposure time (ET), in exposed and unexposed individuals. (A and B) Exposed Miners group. (C) Miner control group. (D) Exposed Residents.
