# Supplementary figures and images for: Unraveling chromosomal and genotoxic damage in individuals occupationally exposed to coal from underground mining
Source: Front Genet. 2024 Jul 4;15:1422938. doi: 10.3389/fgene.2024.1422938 (PMC11254797; doi:10.3389/fgene.2024.1422938)

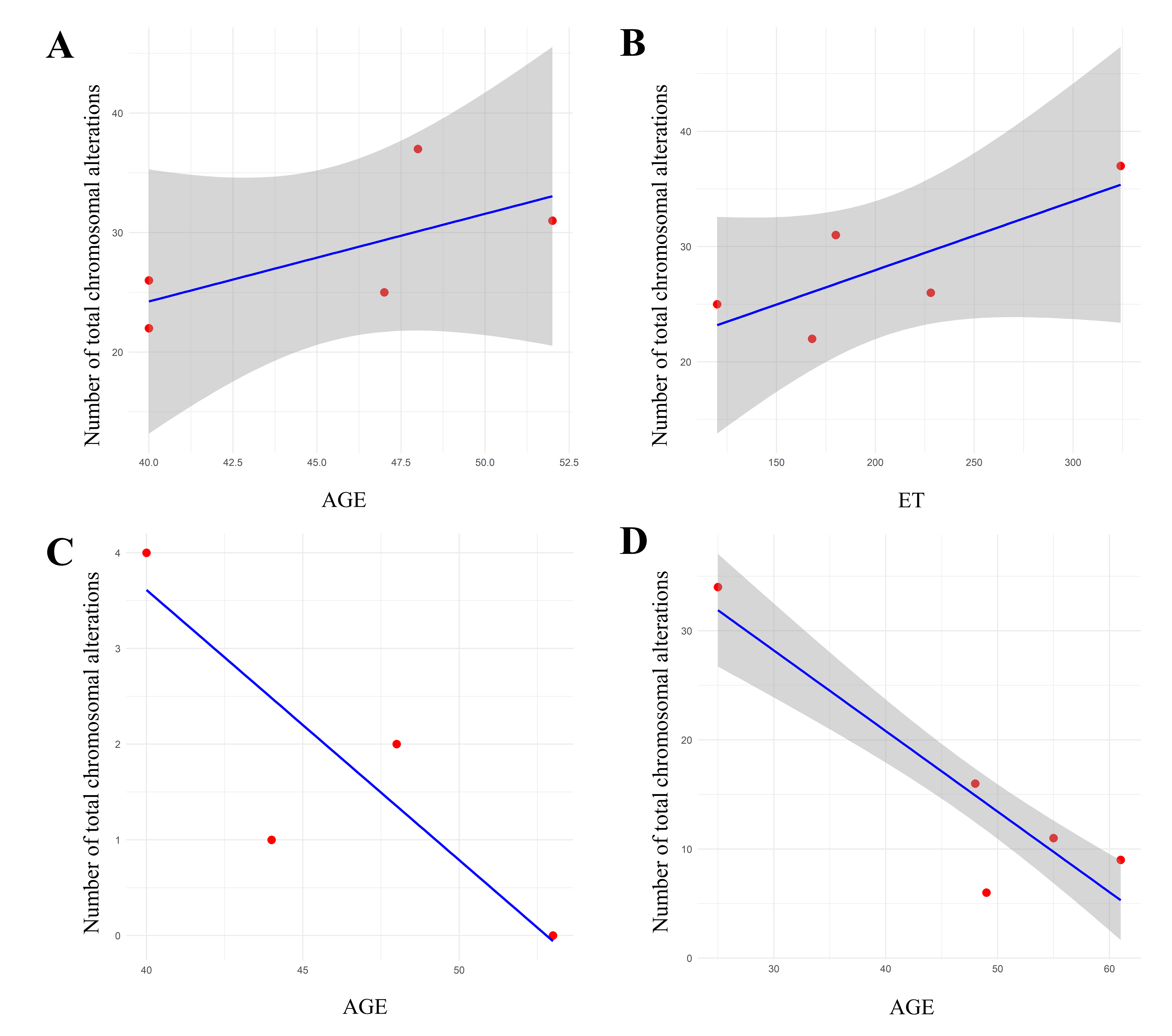

Supplement: Supplementary file 2 [file Image2.tif]

**A**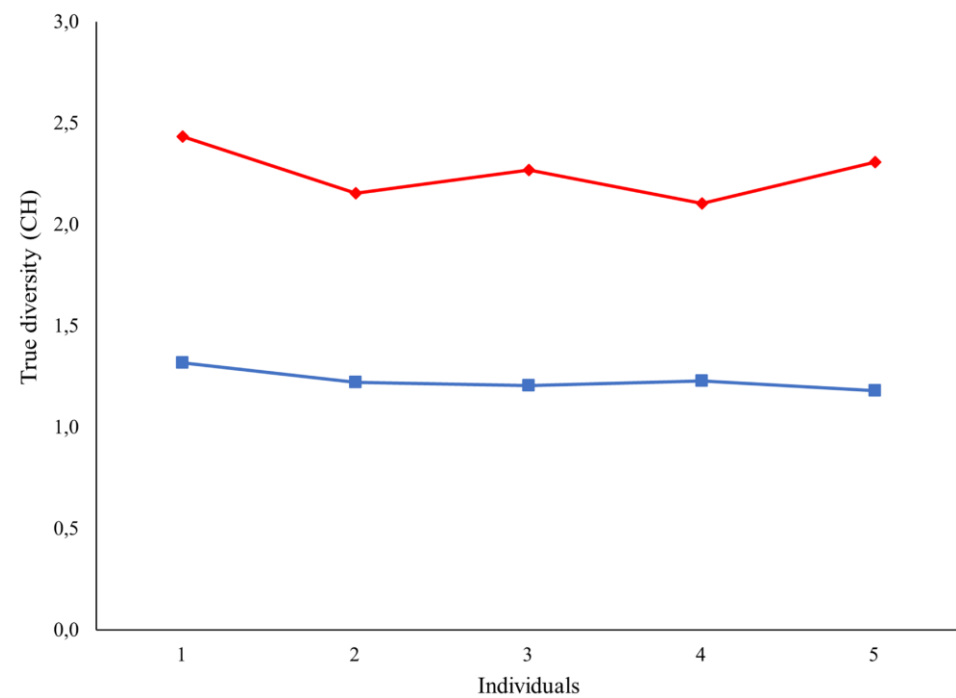**B**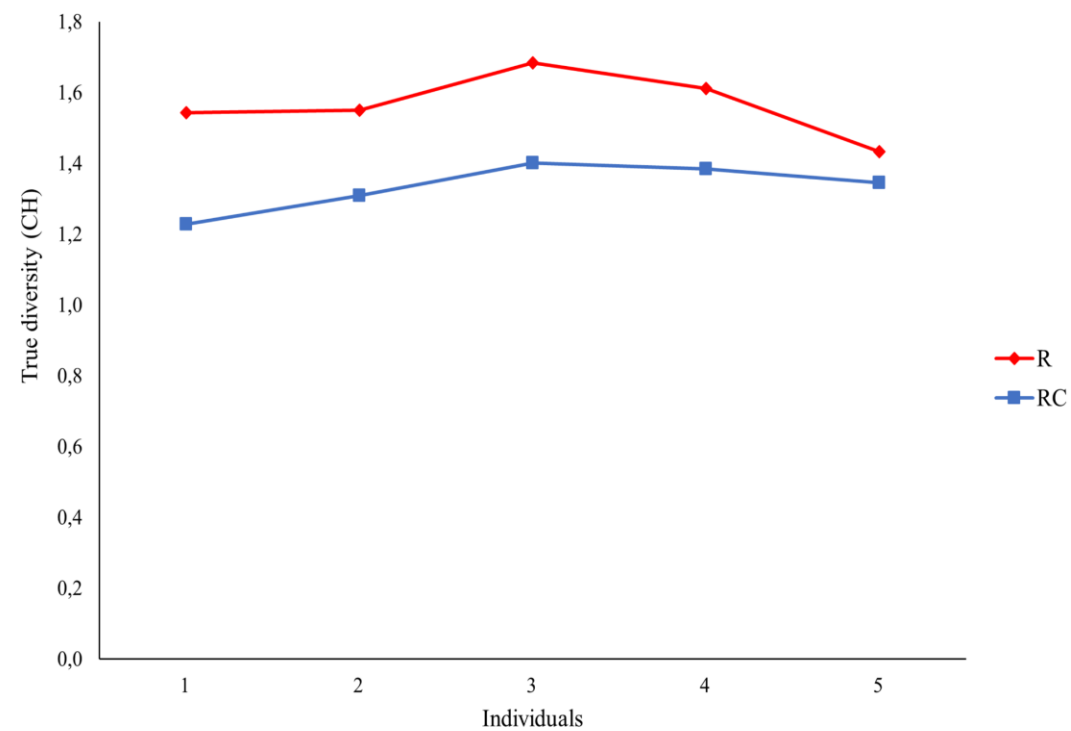

Supplement: Supplementary file 3 [file Image1.pdf]
